# Heavy metal ecological-health risk assessment under wheat–maize rotation system in a high geological background area in eastern China

**DOI:** 10.1038/s41598-022-22608-z

**Published:** 2022-10-26

**Authors:** Fang Wan, Nan Jiang, Linsong Yu, Kai Zang, Shuming Liu, Wei He, Zunfang Hu, Haiyin Fan, Haosong Li, Hui Wang, Hong Pan, Quangang Yang, Yanhong Lou, Yuping Zhuge

**Affiliations:** 1 National Engineering Research Center for Efficient Utilization of Soil and Fertilizer Resources, College of Resources and Environment, Shandong Agricultural University, No. 61 Daizong Street, Tai’an, Shandong China; 2 Shandong Management University, Jinan, Shandong China; 3 Shandong Provincial Engineering Laboratory for Soil Geochemistry/Shandong Provincial Engineering Research Center for Geological Prospecting, Shandong Institute of Geophysical and Geochemical Exploration, Jinan, Shandong China; 4 No.1 Institute of Geology and Mineral Resources of Shandong Province, Jinan, Shandong China

**Keywords:** Biogeochemistry, Environmental sciences, Risk factors

## Abstract

A high geological background can increase the ecological and health risks associated with crop production; therefore, it is essential to assess the heavy metals and their impact. In this study, ecological and health risk impacts of heavy metal contamination, in combination with positive matrix factorization was assessed for an area with high geological background with wheat–maize cropping system, to provide a quantitative understanding of the effects of heavy metals, enabling its prevention and control. This study revealed that the comprehensive ecological risk (*RI*_*wheat–maize*_) is 56.21 (low), with industries being the biggest contributors (34.22%). Comprehensive health risk (non-carcinogenic) assessment showed that industrial (40.98–49.30%) and natural (23.96–37.64%) factors were the primary (particularly of Cd and Zn) and secondary (particularly of Cr and Ni) contributors, respectively in eastern China. Comprehensive health risk (*HI*_*wheat–maize*_) for children and adults were 0.74 and 0.42, respectively, indicating that non-carcinogenic risks were at an acceptable level. Soil ingestion was the primary pathway for health risks (62.23–73.00%), especially for children. Based on soil heavy metal sources and crop systems, source-ecological risk assessment and source-health risk assessment were used to provided valuable insights on making strategies to protect human health in high geological background areas.

## Introduction

Heavy metals soil pollution is a widespread ecological and environmental problem owing to the ubiquity, toxicity, and persistence of these metals. Heavy metals, such as As, Cd, Cu, Pb, and Zn can affect the physical and chemical properties of soils and nutrient absorption by plants^1^, which can cause biomagnification^2^. Furthermore, excess of heavy metals in agricultural soil pose a direct risk to human health via the food chain causing weakening of bones, dermal problems, and neurological disorders^3^.

The sources of heavy metals in soil can be natural or anthropogenic^4^. Anthropogenic sources includes industrial waste^5,6^, metal smelting, and fertilization^7^, while weathering and leaching processes are important natural factors affecting heavy metal pollution in soil. Considering that geological background levels can vary substantially, heavy metal pollution in high geological background areas has received considerable research attention over the recent years^8,9^. In southwest China, studies have reported the enrichment of heavy metals, especially Cd, in karstic soils^10^; however, a few studies revealed that the hilly areas of Eastern China also have a high geological background.

Heavy metals have been found in soil and crops grown locally in China, which poses ecological and human health risks^11– 14^. Therefore, it is important to identify potential sources of agricultural soil contamination to protect human health. Numerous analytical methods and models, including geographic information systems (GIS), multivariate statistical analysis, and receptor models, although not developed for quantitative source identification were used to roughly identify the number and type of sources^15,16^; however, these tools lack the potential to accurately determine the source contribution. As a quantitative receptor model, positive matrix factorization (PMF) has been widely used to identify sources of soil heavy metal pollution^17,18^; however, owing to errors in the PMF model there is a disparity between the calculated and actual results^19^. Therefore, it is necessary to use a combination of methods to reasonably interpret the PMF results. Thus, in this study PMF based on GIS was used to decrease the uncertainty of the PMF model by improving the rationality of parameter settings. In recent years, the health risks posed by heavy metals in farmland soils have garnered substantial attention from researchers and the general public; however, most studies had conducted either health risk assessments or source identification analysis. Therefore, recent studies have combined qualitative source identification methods with risk assessment models to evaluate the potential ecological^20,21^ and human health risks^22–24^ of heavy metal pollution. Most of these studies have focused on source-quantitative health risk assessments under single cropping types, such as soil–wheat, soil–maize, and soil–rice while a few studies have considered rotation systems in the source apportionment for ecological and health risk assessments^25^. Therefore, in this study, we used an integrated method combining PMF with ecological and health risk models to identify heavy metal sources for soil pollution under wheat–maize cropping systems.

As wheat and maize are staple foods in China, wheat–maize rotation is the main farming system in the study area. Therefore, this study aimed to: (1) determine the contamination level of agricultural soils in an area with high geological background; (2) identify and quantify the sources of soil heavy metals using a PMF model; and (3) quantitatively assess the ecological–health risks from the sources in a wheat–maize cropping system. These results hold value in the mitigation of heavy metal pollution risk under a wheat–maize rotation system in a high geological background area.

## Materials and methods

### Study region

The study area (1091 km^2^) is located in Lai’yang (120°31′–120°59′E, 36°48′–37°09′N) in the eastern coastal region of Shandong Province, China. As a provincial economic development zone, the machinery, automobile, chemical, pharmaceutical, electronic, and information technology industries have developed rapidly in this area. Therefore, soil in this region has likely been exposed to various heavy metal sources, especially industrial wastes, traffic emissions, and other anthropogenic factors, generating health and ecological risks. The landforms in eastern Shandong are primarily hilly, with Mesoproterozoic, Neoproterozoic, and Meso–Cenozoic intrusive rocks and widely distributed ancient Mesozoic strata. Dai et al*.*^26^ indicated that there was a high geological background of Ni, Cu and Cr caused by the weathering of sandstone and mudstone in eastern Shandong. The landforms in the study area are dominated by hills with an altitude of 100 to 300 m. Cretaceous strata are distributed across the central and southern parts of the study area, with the lithological elements primarily including glutenite and sand shale. Archean intrusive rocks and Paleoproterozoic strata are well exposed in the north, where lithological elements include gneiss, diorite, and marble. Quaternary sediments are widely distributed across the central and western parts of the study area. The weathering of rocks mostly forms brown acid stone and acid coarse bone soils. Wheat, maize, peanuts, apples, and pears are widely planted in this area.

### Sample collection and preparation

**Figure 1:**
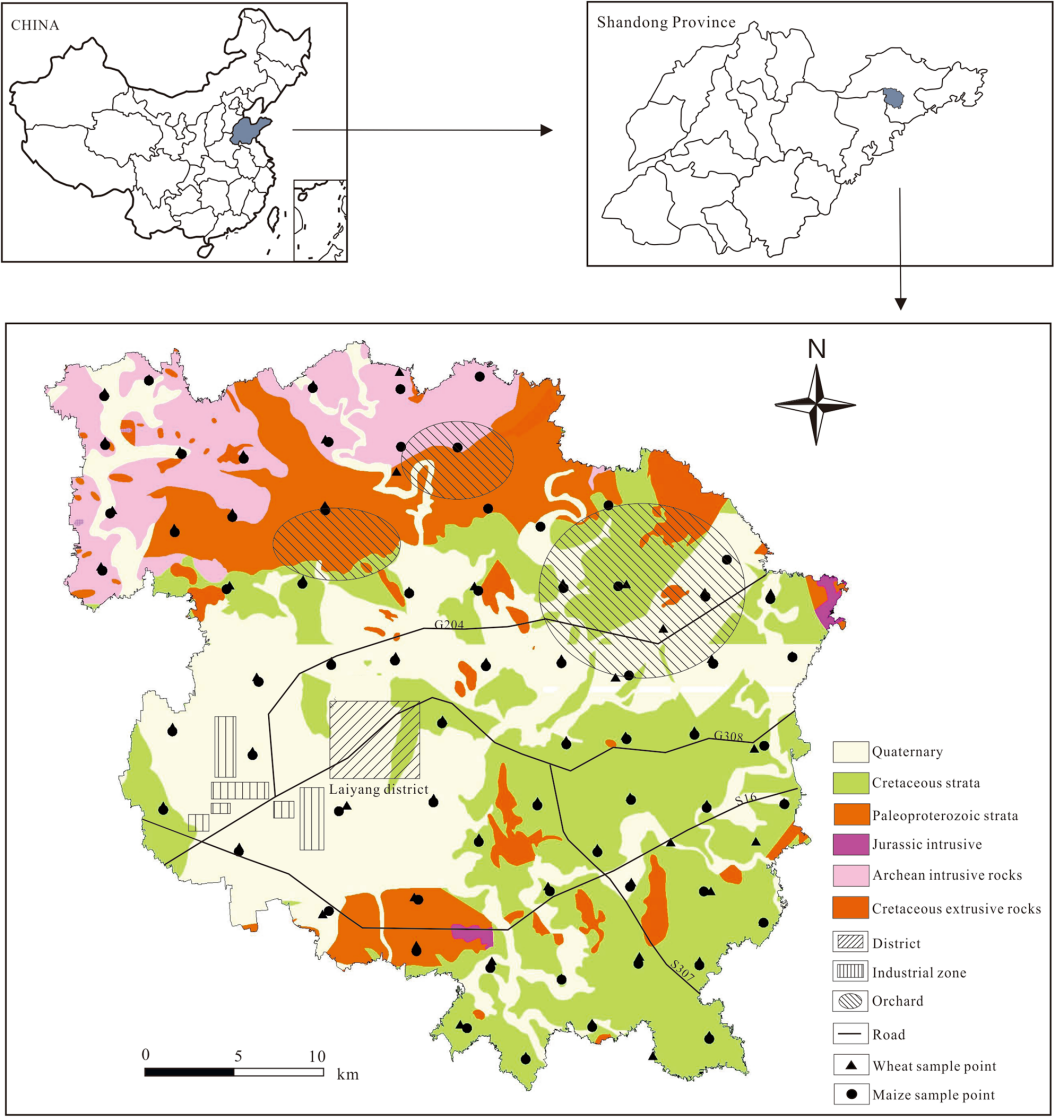
Sampling sites for the present study.

Wheat and maize grain samples (n = 68 each) as well as corresponding 136 soil samples were collected from the study area during their harvest periods in 2019 (Fig. 1). One sample was collected per 16 km^2^, and 1500 g of surface soil (0–20 cm) was collected for each sampling site. The samples were kept in self-sealing polyethylene bags and then transferred to the laboratory.

After removing sand, roots, and other residues, the soil samples were air-dried and passed through a 200-mesh sieve prior to chemical and pH analysis. The grains were washed with deionized water to remove surface dirt, heated at 105 °C for 30 min, and dried to constant weight at 70–80 °C. Thereafter, the dried grains were powdered using an agate grinder and then passed through a 200-mesh sieve before chemical analysis.

### Sample chemical analysis

The concentrations of Cu, Zn, Cr, Ni, and Pb in the soil samples were measured using X-ray fluorescence spectrometry (XRF, PANalytical B.V., Netherlands). To analyze Cd, the samples were digested by HNO_3_-HClO_4_-HF (1:1:2, v/v/v) and analyzed using an inductively coupled plasma mass spectrometer (ICP-MS, Varian 820, USA). The wheat and maize grain samples were digested in aqua regia (HNO_3_:HCl = 3:1). The concentration of Cd, Cu, Zn, Cr, Ni and Pb was determined using ICP-MS (Varian 820, USA) and the method detection limit (MDL) for Cd, Cu, Zn, Cr, Ni and Pb were 0.1 μg/g, 1.0 μg/g, 1.0 μg/g, 0.5 μg/g, 0.1 μg/g and 0.1 μg/g, respectively. Soil pH was measured at a soil:water ratio of 1:2.5 by using a pH meter (PHS-3C, Shanghai, China). All the analysis methods were according to Ministry of Land and Resources of the People's Republic of China: Specification of land quality geochemical assessment (DZ/T 0295-2016).

Quality assurance/quality control (QA/QC) was conducted using blank and replicate samples based on standard reference materials (GBW07412, GBW07417), obtained from the Center of National Standard Reference Material of China. Results showed that the relative deviation of duplicate samples for Cd, Cu, Zn, Cr, Ni and Pb were 94.8%, 100%, 100%, 100%, 98.5% and 100%, respectively. The relative standard deviation (RSD) and logarithmic deviation (ΔlogC) were both within 6%. The recovery values for Cd, Cu, Zn, Cr, Ni and Pb were 92.3–110.2%, 94.5–98.1%, 96.0–100.1%, 98.0–102.8%, 94.5–102.5% and 97.0–100.2%, respectively.

### Geoaccumulation index (I_geo_)

The *I*_*geo*_ was used to evaluate soil metal pollution using Eq. (1):^27^

1 $${I}_{geo}={log}_{2}\frac{{C}_{n}}{1.5{B}_{n}}$$

where *C*_*n*_ was the concentration of metal (*n*) in the soil sample, and *B*_*n*_ was the geochemical background concentration of metal (*n*) adopted by the background values of soil in Shandong Province^28^. Because of lithologic variation in the soil, a value of 1.5 was used as the background matrix correction factor^29^. The *I*_*geo*_ comprised seven classes^30^, and the evaluated standards for *I*_*geo*_ with the corresponding classes are shown in Table S1.

### PMF model

PMF is a useful analysis model for source apportionment^31^. In the current study, PMF 5.0 of the United States Environmental Protection Agency (USEPA) was used to identify the source profile and contribution. The main principle of this method is to decompose the original matrix *x*_*ij*_ into matrices *g*_*ik*_ and *f*_*jk*_ as well as a residual matrix *e*_*ij*_, as shown in Eq. (2):

2 $$x_{ij} = \mathop \sum \limits_{k = 1}^{p} g_{ik} f_{kj} + e_{ij} ,$$

where *x*_*ij*_ was the concentration of heavy metal *j* in sample *i*, *p* was the number of sources, *g*_*ij*_ was the contribution of factor *k* to sample *i*, *f*_*kj*_ was the concentration of heavy metal *j* in source *k*, and *e*_*ij*_ was the residual error matrix. The PMF model minimized the value of the objective function *Q*, as shown in Eq. (3):

3 $$Q = \mathop \sum \limits_{i = 1}^{n} \mathop \sum \limits_{j = 1}^{m} \left( {\frac{{e_{ij} }}{{u_{ij} }}} \right)^{2} ,$$

where *u*_*ij*_ was the uncertainty of heavy metal *j* in sample *i*. If the concentration of heavy metal was greater than its corresponding minimum detection limit (*MDL*) value, the uncertainty was calculated as shown in Eq. (4). Otherwise, it was calculated using Eq. (5):

4 $$Unc = \sqrt {\left( {Errofraction \times concenration} \right)^{2} + \left( {0.5 \times MDL} \right)^{2} } ,\;\;{\text{or}}$$

5 $$Unc = \frac{5}{6} \times MDL.$$

### PMF-based ecological risk model

The ecological risk index (*RI*) was applied to evaluate the degree of ecological risk caused by soil heavy metals in the study area. The *RI* results indicate the toxicological effects of heavy metals and the associated environmental response^32^. The PMF-based *RI* model was developed using the receptor model and the ecological risk assessment of soil heavy metals. It is a new integrated method used to quantify the ecological risks of soil heavy metals from different sources in wheat–maize cropping patterns. The source contribution of heavy metals in each sample was estimated by using the PMF 5.0 model as follows:

6 $$C_{jn}^{l} = *C_{jn}^{l} \times C_{j} ,$$

where *C*^*l*^_*jn*_ was the mass contribution (mg·kg^−1^) of heavy metal *n* from source *l* in sample *j*; *C^l^_jn_ was the calculated contribution of heavy metal (*n*) from source *l* in sample *j*; and *C*_*j*_ was the concentration (mg·kg^−1^) of soil heavy metals in sample *j*. Equation (6) was used to quantify the ecological risk of soil heavy metals from different sources in wheat–maize rotation cropping systems. The ecological risk of heavy metals from source *l* in sample *j* was calculated using Eq. (7):

7 $$RI_{j}^{l} = \sum( E_{r}^{i} )_{j}^{l} = \sum \frac{{C_{jn}^{l} }}{{B_{i} }} \times T_{r}^{i} ,$$

where (*E*^*i*^_*r*_)^*l*^_*j*_ was the calculated ecological risk of each heavy metal from source *l* in sample *j*; *B*_*i*_ was the background value; and *T*^*i*^_*r*_ was the toxicity response coefficient for a given heavy metal *i*, and accounts for toxicity and sensitivity requirements (Zn: 1; Cu: 5; Pb: 5; Cr: 2; Cd: 30)^32^. The evaluated standards for the ecological risk and the corresponding grades for *E*^*i*^_*r*_ and *RI* are given in Table S2^30^.

### PMF-based health risk model

The health risk assessment and PMF were combined to quantitatively determine the contribution of the health risk from heavy metal sources under wheat and maize cropping systems. The health risk model of USEPA was used to calculate the non-carcinogenic risk. Similar to the PMF-based ecological risk model, the PMF-based health risk model had two steps. First, the source contribution of heavy metals in each soil sample was calculated using Eq. (6). Thereafter, the health risks posed by the heavy metals from different sources were quantitatively characterized for different cropping systems. The average daily exposure doses (*ADD*^*l*^_*jn,i*_) for heavy metals from four exposure pathways *i*, namely soil ingestion, inhalation via nose and mouth, dermal contact, and food ingestion, from source *l* of the heavy metal *n* in sample *j* was calculated using the Eqs. (8–11):

8 $${ADD}_{jn,ing}^{l}= \frac{{C}_{jn}^{l} \times {IR}_{ing} \times EF \times ED}{BW \times AT} \times {10}^{-6}$$

9 $${ADD}_{jn.inh}^{l}= \frac{{C}_{jn}^{l} \times {IR}_{inh} \times EF \times ED}{PEF \times BW \times AT}$$

10 $${ADD}_{jn.dermal}^{l}= \frac{{C}_{jn}^{l} \times SA \times AF \times ABS \times EF \times ED}{BW \times AT} \times {10}^{-6}$$

11 $${ADD}_{jn.diet}^{l}= \frac{{C}_{jn}^{l} \times {IR}_{diet} \times EF \times ED}{BW \times AT} \times {10}^{-6}$$

where *C*^*l*^_*jn*_ was the concentration of the *j*-th metal in the *n*-th sample from the *l*-th source (mg·kg^−1^ ·day^−1^); IR_*ing*_, IR_*inh*_, and IR_*diet*_ were the ingestion rate through soil ingestion (mg·day^−1^), soil inhalation (m^3^·day^−1^), and food ingestion (mg·day^−1^), respectively; *SA* was the exposed surface area of the skin (cm^2^); *AF* was the adherence factor (kg·cm^−2^·day^−1^); *ABS* was the dermal absorption factor (unitless); *PEF* was the emission factor (m^3^·kg^−1^); *EF* was the exposure frequency (d·y^−1^); *ED* was the exposure duration (y); *BW* was the body weight of the exposed individual (kg); and *AT* was the average time of exposure to contaminated soils (d), with 10^−6^ being a unit conversion factor. Details of the parameters that were applied to the exposure assessment model are given in Table S3.

The non-carcinogenic hazard of heavy metals can be expressed using the hazard quotient (HQ), which is the quotient of the ADD of each heavy metal to the corresponding reference dose (RfD). The RfD of each heavy metal is shown in Table S4. *HQ*^*l*^_*jn,i*_ is the hazard quotient through the *i*-th exposure pathway from source *l* of heavy metal *n* in sample *j*. The hazard index (*HI*) was calculated using Eq. (12):

12 $${HI}_{jn}^{l}= \sum {HQ}_{jn,i}^{l}= \sum \frac{{ADD}_{jn,i}^{l}}{{RfD}_{i}}$$

### Data analyse

Descriptive statistical analyses were conducted using SPSS 22.0 (IBM, USA). Figure 1 was generated using ArcGIS 10.2 (Esri, USA, http://resources.arcgis.com/en/help/install-guides/arcgis-server/10.2/). Figure 3 and Figure S3 were generated using GeoIPAS V4.2 (JWSOFT, China, https://www.jinweisoft.com/).

### Ethics approval

Soil and plant samples collection were complied with relevant institutional, national, and international guidelines and legislation.

## Results and discussion

### Heavy metal concentration in the soil samples

The descriptive statistics for heavy metal concentrations in the soil samples are shown in Table 1, with the concentrations in the order of Cr > Zn > Ni > Cu > Pb > Cd for both wheat and maize cropping systems. In the current study, average concentrations of Cr, Cu, and Ni were higher than those for the soil background values in China^33^. The maximum concentrations of Cd, Cr, and Ni were 4.63-, 6.14-, and 4.74-times of their background values in China. Additionally, the mean concentrations of Cr, Cu, and Ni were higher than the background values from surface soil in the Shandong Province, China^28^ and the U.S.A^34,35^. According to the Environmental Protection Administration of China (EPAC), the excess rate of soil samples was 12.41%. Notably, 2.94% of soil samples exceeded the standard values for agricultural soils in China for Cr and Ni in wheat cropping systems and a total of 4.41, 5.88, and 5.88% of soil samples from maize cropping systems exceeded the standard values for Cd, Cr, and Ni, respectively. The average value of pH of the soil samples was less than 6.5, indicating soil acidification, which makes the crops prone to heavy metal toxicity^36^. According to the National Health and Family Planning Commission of the People’s Republic of China and the China Food and Drug Administration (NHFPCPRC and CFDA: GB2762-2017, 2017), the excess rates of Pb and Cr in maize samples were 4.41% and 11.76% (Table S5). Notably, three and eight maize samples exceeded the limits of Pb and Cr, respectively. In contrast, all the wheat samples were below the limits. The results indicate some of the sites were contaminated by heavy metals, where health risk assessments should be focused.

**Table 1:** Statistical characteristics of soil heavy metals in wheat–maize cropping systems (mg·kg^-1^).

HM	Wheat cropping system (n = 68)					Maize cropping system (n = 68)					BV^a^	BV^b^	BV^c^	SV^d^
	Mean	Min	Max	SD	CV%	Mean	Min	Max	SD	CV%				pH ≤ 5.5/ 5.5 < pH ≤ 6.5/ 6.5 < pH ≤ 7.5
Cd	0.10	0.04	0.27	0.04	35.22%	0.12	0.03	0.45	0.07	62.82%	0.097	0.13	0.18	0.3/0.3/0.3
Cr	85.03	16.45	330.55	48.01	56.46%	96.51	16.08	380.71	62.95	65.23%	61.0	61.0	37	150/150/200
Cu	28.57	11.36	73.00	13.61	47.62%	27.79	10.98	92.79	12.37	44.51%	22.6	22.6	17	50/50/100
Ni	30.58	8.30	127.48	16.49	53.91%	34.23	8.11	114.44	20.31	59.33%	26.9	27.1	13	60/70/100
Pb	22.64	9.67	37.29	5.07	22.40%	21.65	6.57	36.70	5.18	23.91%	26.0	23.6	16	70/90/120
Zn	42.96	18.97	92.95	19.21	44.85%	64.96	34.69	117.64	17.22	26.51%	74.2	63.3	48	200/200/250
pH	6.23	5.16	7.14	0.48	7.68	6.18	5.18	7.34	0.55	8.88%	7.7	7.32		

HM = heavy metals; SD = standard deviation; CV = coefficient of variance; BV = background value; SV = standard value.

^a^CNEMC (China National Environmental Monitoring Center) 1990. Soil Element Background Values in China. China Environmental Science Press, Beijing, China.

^b^Background Values of Soil in Shandong Province^24^.

^c^Background Values of Soil in United States of America^28,29^.

^d^Environmental Protection Administration of China (EPAC; GB15618-2018).

### Assessment of heavy metal accumulation and pollution

The *I*_*geo*_ was also used as a reference for estimating the extent of metal pollution (Table 2). The mean *I*_*geo*_ values were below zero for each heavy metal under both wheat and maize cropping systems, indicating that the soil is generally uncontaminated in the study area. It is important to note that more than 76 and 80% of the soil samples were found to be free from contamination in the maize and wheat cropping systems, respectively. Moreover, 2.94% of the soil samples (2 samples) were moderately contaminated with Cr, Cu, and Ni in the wheat cropping system, respectively. A total of 1.47, 4.41, 1.47, and 4.41% of the soil samples were moderately contaminated with Cd, Cr, Cu, and Ni in the maize cropping system, respectively. One soil sample was moderately to heavily contaminated with Cr in the maize cropping system. Therefore, it was concluded that while the areas in the study region are mainly uncontaminated, some points had moderate contamination of heavy metals, especially with respect to Cr, Cu, and Ni.

**Table 2:** Pollution classification of soil heavy metals in wheat and maize cropping systems.

Cropping system	Pollution classification of *I*_*geo*_		Cd	Cr	Cu	Ni	Pb	Zn
Wheat n = 68	percentage of the samples in each class (%)	Unpolluted	98.53	80.88	80.88	89.71	98.53	100
		Unpolluted to moderately contaminated	1.47	16.18	16.18	7.35	1.47	
		Moderately contaminated		2.94	2.94	2.94		
	Mean *I*_*geo*_		− 1.05	− 0.28	− 0.37	− 0.54	− 0.68	− 1.27
Maize n = 68	percentage of the samples in each class (%)	Unpolluted	91.18	76.47	80.88	85.29	98.53	95.59
		Unpolluted to moderately contaminated	7.35	17.65	17.65	10.29	1.47	4.41
		Moderately contaminated	1.47	4.41	1.47	4.41		
		Moderately to heavily contaminated		1.47				
	Mean I_geo_		− 0.97	− 0.13	− 0.39	− 0.41	− 0.76	− 0.60

### Source apportionment in wheat–maize rotation systems

**Figure 2:**
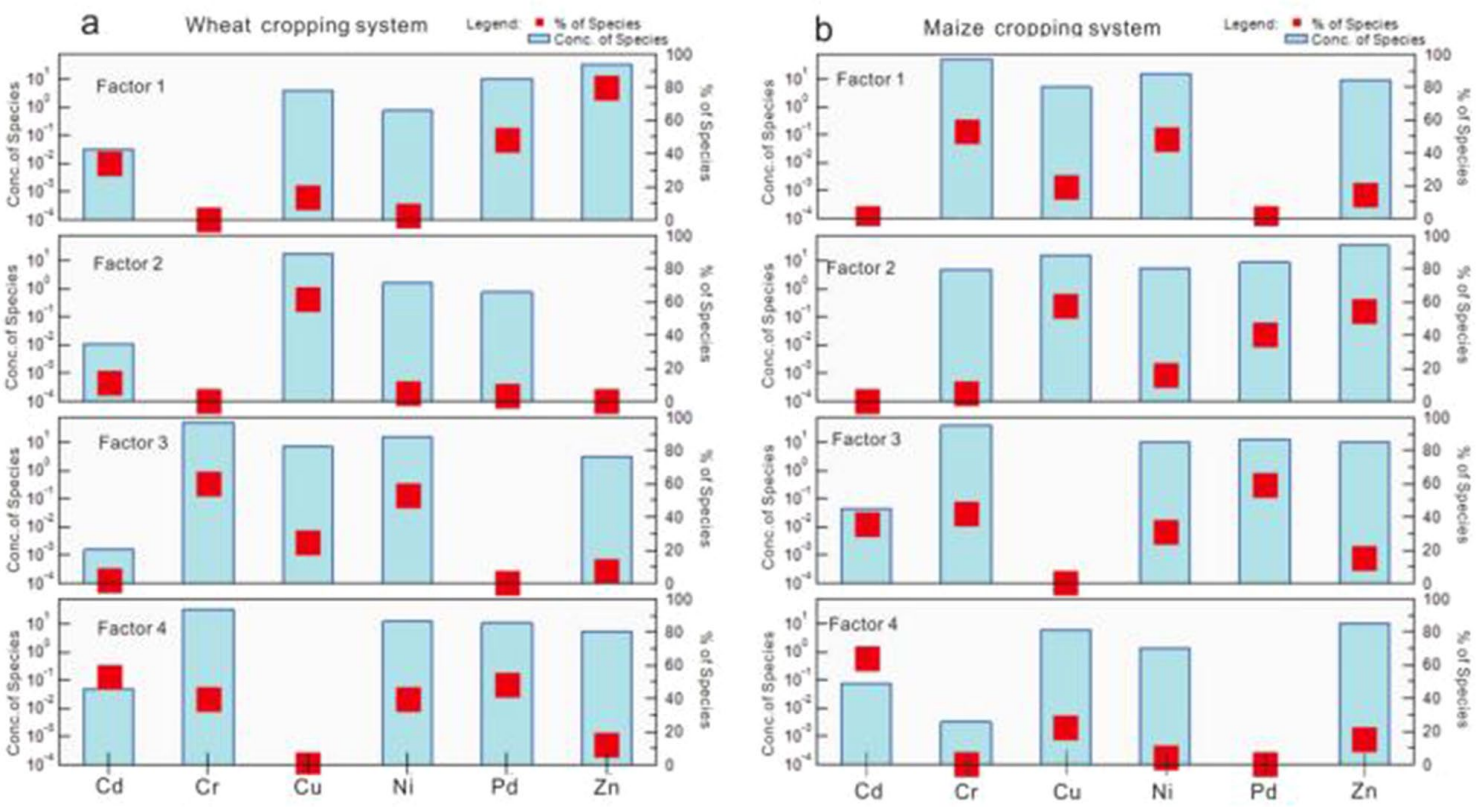
Factor profile of heavy metals in (**a**) wheat and (**b**) maize cropping systems based on a positive matrix factorization model.

**Figure 3:**
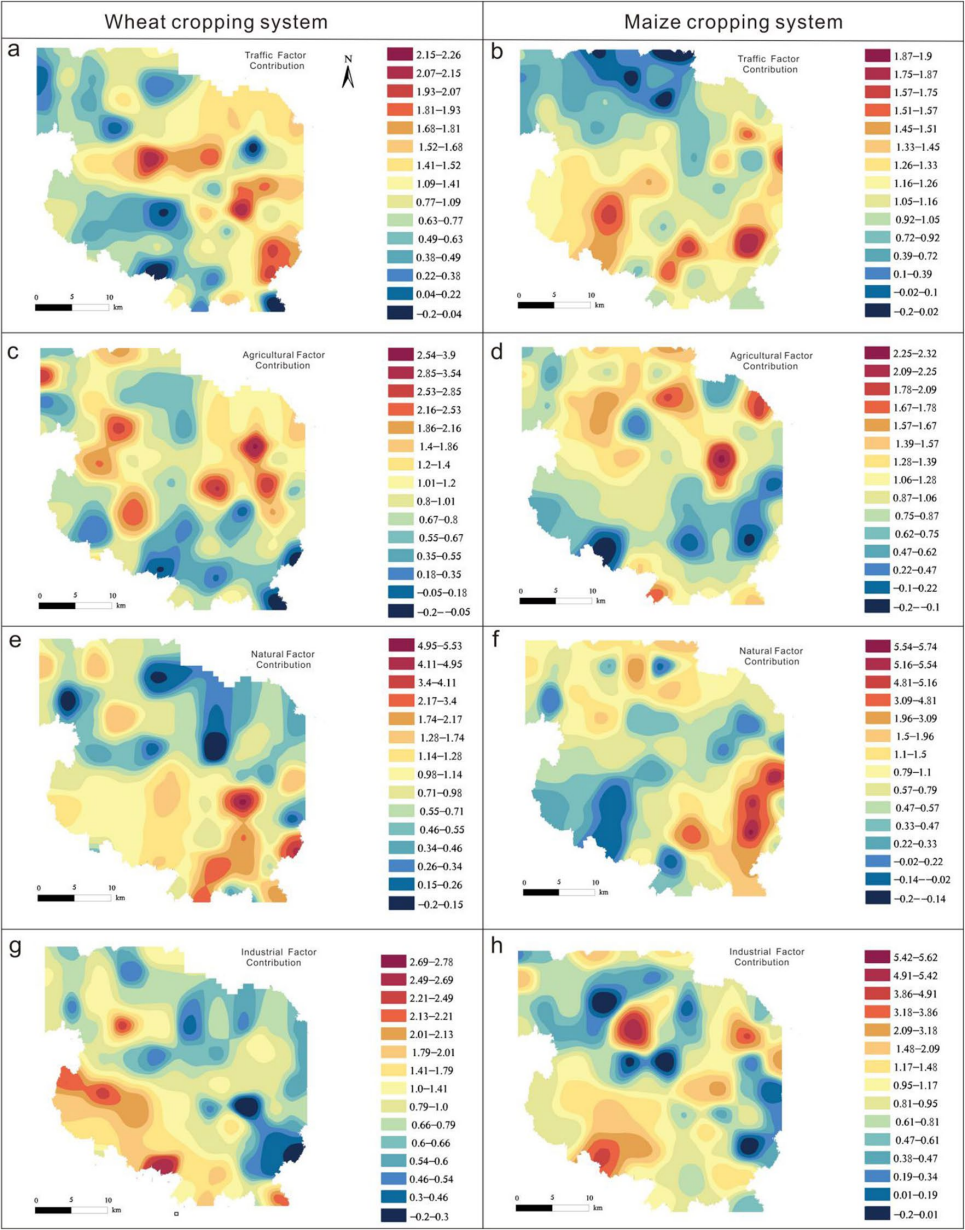
Spatial distribution of the normalized contributions (average of all sample sites = 1).

The PMF 5.0 model was used to identify the source categories and quantify the source contributions. The start seed number was chosen at random, and the number of runs was set to 20. When the number of source factors was 4, the *Q* value was at the minimum and stable. The signal-to-noise (S/N) ratios for the six heavy metals were strong, and the absolute scaled residuals were acceptable. The concentration of species and the source contributions are shown in Fig. 2. Additionally, GIS was used to determine the spatial distribution of the normalized contribution of each factor, as shown in Fig. 3.

In the wheat cropping soils, factor 4 (32.21% of the total sources) had a strong positive loading of Cd (52.77%) and Pb (48.52%), whereas in the maize cropping soils, factor 3 (29.75% of the total sources) showed strong positive loading of Pb (60.28%) and Cr (43.78%) (Fig. 2). The high values for areas with overlapping normalized contributions for wheat and maize cropping systems indicated that these areas were most affected by a specific source. The spatial distribution of the normalized contribution for both wheat and maize cropping systems indicated that higher values were primarily observed in the southeastern part of the study area around the S307 provincial road (Fig. 3a, b). According to previous studies, Cd and Pb accumulate in the roadside soil owing to atmospheric deposition related to vehicle emissions^37,38^. Cr is also associated with traffic emissions^39,40^. Therefore, these factors were classified as being from a traffic source.

In the wheat cropping system, factor 2 accounted for 13.64% of the total contribution of the selected heavy metals, which was dominated by Cu (62.10%); however, in the maize cropping system, factor 2 accounted for 27.57% of the total contribution, dominated by Cu (59.47%) and Zn (53.83%). In addition, the spatial distribution pattern of the normalized contribution of factor 2 for both wheat and maize cropping systems revealed that the overlap of high value areas was primarily in orchards and other cultivated land in the central eastern section of the study area (Fig. 3c, d). Previous studies have reported that long-standing farming practices, such as the use of pesticides, fertilizers, and livestock manure, have contributed to the high concentrations of Cu and Zn in the soil^41,42^. Therefore, factor 2 was regarded as derived from the agricultural sector.

In the wheat cropping system, factor 3 accounted for 24.42% of the total contribution, dominated by Cr (59.97%) and Ni (52.79%), whereas in the maize cropping system, factor 1 accounted for 24.41% of the total contribution, dominated by Cr (53.57%) and Ni (49.00%). The spatial distribution of the normalized contribution of factor 3 and 1 for wheat and maize cropping system, respectively, showed that overlapping areas with high values were primarily located at the southeastern section of the study area (Fig. 3e, f), which coincided with the distribution of Cretaceous strata. Previous studies have shown that high concentrations of Ni and Cr are predominantly present in the natural background, which is controlled by weathering of parent material, as well as pedogenesis^7^. Liu et al*.*^9^ reported that the Ni and Cr present in agricultural soils in the southern Shandong peninsula primarily originated from natural sources. Therefore, factor 3 and factor 1in the wheat and maize cropping system were classified as derived from natural sources.

For heavy metals in wheat cropping soils, factor 1 (29.72% of the total sources) had a strong positive loading of Zn (79.95%) and Pb (48.07%), while in maize cropping soils, factor 4 (18.28% of the total source) showed strong positive loading of Cd (62.77%). The spatial distribution of the normalized contribution of factor 1 in the wheat cropping system and factor 4 in the maize cropping system indicated that the overlapping high values were primarily in the southwestern section of the study area, which is adjacent to an industrial zone (Fig. 3g, h). According to previous studies, the presence of chemical plants is closely associated with a high concentration of Cd, Pb, and Zn^43^. Wang et al*.*^44^ also reported that Cd pollution primarily originated from industrial and traffic sources. Therefore, factors 1 and 4 were regarded to be predominantly derived from industrial activities.

### Quantification of ecological risks in crop rotation systems

In the current study, a PMF-based ecological risk model was established to quantify the ecological risk of heavy metals in soil from four identified sources. *RI* (wheat–maize) means the average value of *RI* (wheat) and *RI* (maize), representing the whole ecological risk in wheat–maize rotation system. As shown in Table 3, the mean potential ecological risk for soil heavy metals in wheat–maize rotation systems was 56.21. This indicated that the potential ecological risks of soil heavy metals were low, and industrial factors had the highest contribution with 34.22%. Furthermore, the ecological risk from different sources varied with the cropping systems. For the wheat cropping system, the order of contribution of sources was traffic (27.30%) > agriculture (24.80%) > industries (24.33%) > nature (23.57%), whereas in the maize cropping system, the order of contribution was industry (35.61%) > traffic (24.55%) > agriculture (21.50%) > nature (18.34%). Further, the potential ecological risk was slightly higher for maize than the wheat cropping system. This was because soil heavy metals were more affected by industrial activities during the growing stage of the maize. Generally, heavy metals closely related to industrial emissions are highly toxic. A prior study indicated that industrial influences had the highest ecological risk between natural sources, traffic emissions, and agricultural practices in Guangdong Province, southeast China^17^. Owing to the high toxicity response coefficients for Cd, the industrial source for the maize cropping system should be prioritized for supervision and management to reduce the potential ecological risks.

**Table 3:** Ecological risks from soil heavy metals in wheat and maize cropping systems.

Cropping system	Traffic source	Agricultural source	Industrial source	Natural source	Total
Wheat cropping system	11.82	10.73	10.53	10.20	43.28
Proportion	27.30%	24.80%	24.33%	23.57%	100.00%
Maize cropping system	19.26	16.87	27.94	14.39	69.13
Proportion	24.55%	21.50%	35.61%	18.34%	100.00%
Wheat–Maize	15.54	13.80	19.24	12.30	56.21
Proportion	27.65%	24.55%	34.22%	21.87%	100.00%

### Health risk assessment

The normalized source contribution for soil heavy metals was obtained using the PMF model (Figure S1). This model showed the contribution of source factors to the total mass for each sample. According to the mass contribution from the samples, the multiple health risk values of the heavy metals in each sample were obtained. The health risk values of different sources for adults and children in wheat and maize cropping systems are shown in Table 4. The non-carcinogenic health risk (*HI*s) for children and adults under the wheat and maize cropping systems were all less than 1. In order to evaluate the comprehensive health risk in wheat–maize rotation system, *HI*^*l*^_*j,r*_ was introduced using Eq. (13):

**Table 4:** Non-carcinogenic risks for wheat and maize cropping systems.

Cropping system	Non-cancer risk in children					Non-cancer risk in adults				
	Natural Factors	Traffic Factors	Industrial Factors	Agricultural Factors	Total	Natural Factors	Traffic Factors	Industrial Factors	Agricultural Factors	Total
Wheat	7.01E−02	2.64E−02	5.79E−02	3.09E−02	1.85E−01	3.41E−02	1.29E−02	2.82E−02	1.50E−02	9.02E−02
Proportion	37.84%	14.26%	31.24%	16.67%	100%	37.84%	14.26%	31.24%	16.67%	100%
Maize	4.87E−02	5.73E−02	6.24E−02	5.73E−02	2.26E−01	3.02E−02	2.90E−02	3.00E−02	2.59E−02	1.15E−01
Proportion	21.60%	25.38%	27.65%	25.38%	100%	26.25%	25.18%	26.06%	22.52%	100%
Wheat–Maize	1.77E−01	1.02E−01	3.63E−01	9.48E−02	7.37E−01	1.57E−01	5.34E−02	1.71E−01	3.57E−02	4.17E−01
Proportion	23.96%	13.89%	49.30%	12.86%	100%	37.64%	12.81%	40.98%	8.57%	100%

13 $${HI}_{j,r}^{l}= \sqrt{\frac{{{({HI}_{j,r}^{l})}_{average}}^{2}+ {{({HI}_{j,r}^{l})}_{maximum}}^{2}}{2}}$$

where *HI*^*l*^_*j,r*_ is the hazard quotient under rotation system *r* from source *l* in sample *j*. The highest comprehensive *HI* value was 0.74 for children under wheat–maize rotation system (Table 4), indicating a low non-carcinogenic risk in the high geological background area. Specifically, the *HQ* for the selected heavy metals in the maize cropping system were in the order of Pb > Cr > Ni > Cu > Zn > Cd for children and Cd > Cr > Pb > Ni > Cu > Zn for adults. However, the *HQ* values for all heavy metals were less than 1 (Figure S2). The health risk based on source apportion decreased in the following order: industrial factors (40.98–49.30%) > natural factors (23.96–37.64%) > traffic factors (12.81–13.89%) > agricultural factors (8.57–12.86%). Changes in the *HI* contribution from each source factor in the wheat–maize system are shown in Table 4. The order of contribution for children and adults from the wheat cropping systems was nature (37.84%) > industries (31.24%) > agriculture (16.67%) > traffic (14.26%), while that for adults and children from the maize cropping systems was nature (26.25%) > industries (26.06%) > traffic (25.16%) > agriculture (22.52%) and industry (27.65%) > traffic (25.38%) = agriculture (25.38%) > nature (21.60%), respectively.

The spatial distribution of *HI* provides the geographic information of the high health risk locations. As is shown in Figure S3, the high value areas are concentrated in hilly agricultural land located at the southeastern section of the study area in wheat cropping system (Figure S3a,b) and were primarily in orchards at the central eastern section of the study area under maize cropping system(Figure S3c,d). The spatial distribution of health risks is closely related to the distribution of heavy metals in high geological background areas. Tang et al*.*^45^ reported a high geological background of heavy metals (As and Cd) in soils with carbonate rocks from the Devonian to Permian periods in southwestern China, which posed non-carcinogenic risks.

Owing to the high mobility and bioavailability of Cd, the high concentration of geological background Cd in soil–rice cropping systems in the karst plains and hilly areas in south China pose a great health risk for local residents. In contrast with the high geological background areas in southwestern China, the high geological background areas in Eastern China pose a low human health risk. Two primary reasons for this are: (1) the high geological background elements in southwestern China are mainly Cd, while those in Eastern China are mainly Cr, and the toxicity of Cd is much higher than that of Cr and (2) rice is mainly planted in the high geological background areas in southwestern China, while wheat and maize are the main crops in Eastern China, and the enrichment ability of rice to heavy metals is higher than that of wheat and maize^46^.

In the study location, wheat and maize are also cultivated in an area with a high geological background of heavy metals (Cr and Ni) in Eastern China. The calculated *I*_*geo*_ revealed a higher environmental risk for Cr and Ni than for other heavy metals in this area. In general, Cr is primarily trivalent and has less mobility and toxicity. Under the high background of Cr in the study area with low pH, the migration of Cr is high from soil to maize, causing some maize samples to exceed the limit. As maize is largely used in animal feed and industrial raw materials, the ingestion rate of maize was far below that of wheat^25^. Thus, the HQ of Cr through diet ingestion was much lower (Figure S2). Therefore, Cr and Ni in the soil–wheat/maize systems in the high geological background area of Eastern China have negligible human health risks. However, the Cd from industrial sources in the research area requires attention.

**Figure 4:**
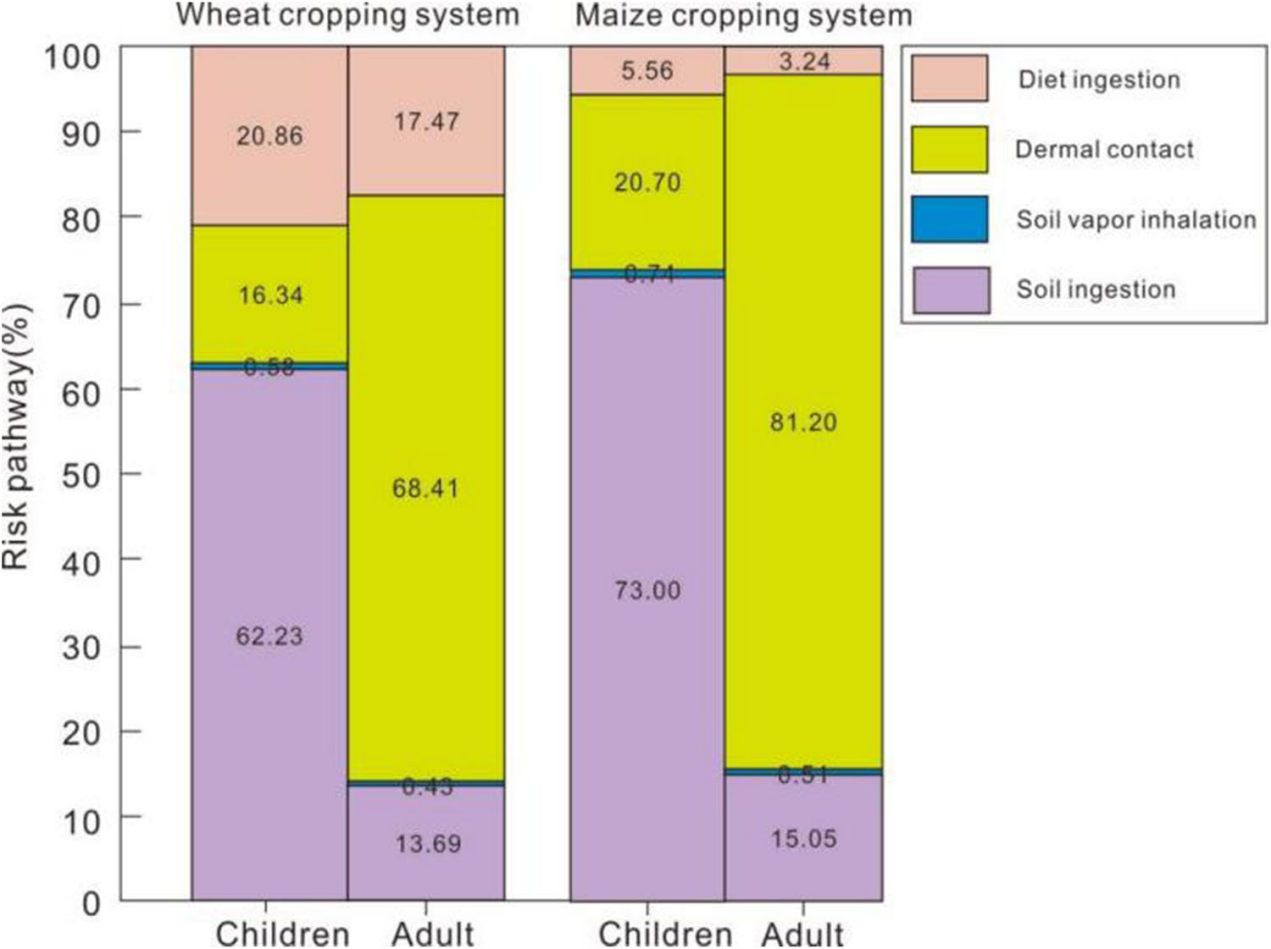
Non-carcinogenic risk exposure pathways under wheat and maize cropping systems.

Heavy metals accumulate in the human body primarily through soil ingestion, food ingestion, dermal contact, and soil vapor inhalation. Thus, it is important to identify the contribution of each exposure pathway. The results of our study showed that exposure pathways for children and adults varied greatly. For children, soil ingestion was the main exposure pathway accounting for 62.23 and 73.00% of the potential health risks for wheat and maize cropping systems, respectively (Fig. 4). while for adults, the pathway was soil dermal contact (68.41–81.20%) and soil ingestion (13.69–15.05%) in wheat–maize cropping systems. Due to insufficient data and interactions of metals, the health risks through soil ingestion may be underestimated for adults^47^. Therefore, it is considered that soil ingestion is the most important route of exposure^48^ and high geological background areas further increase the health risks for children due to soil intake^49^. The health risks associated with the consumption of wheat and maize products in this area are relatively low.

## Conclusion

In this study, ecological and health risk models based on PMF indicated that the average concentrations of Cr, Cu, and Ni in the study area soil were higher than the background soil values for China and the U.S.A. Traffic, agriculture, nature, and industry were identified as the sources of six heavy metals, and the potential ecological risk from these sources were low. The comprehensive health risk (*HI*_wheat–maize_) of 0.74 (children) and 0.42 (adults) indicates that there was no non-carcinogenic risk in the high geological background area in Eastern China, and the health risk based on PMF decreased in the following order: industrial factors (40.98–49.30%) > natural factors (23.96–37.64%) > traffic factors (12.81–13.89%) > agricultural factors (8.57–12.86%). Furthermore, The ecological risks and human health risks caused by industrial factor were greater than those caused by natural factor in the high geological background areas of eastern China. Findings from the current study have provided an effective approach for risk apportionment, which is of great significance for pollution control and risk reduction under rotation cropping systems in high geological background areas.

## Supplementary Information

Supplementary Information.

## Data Availability

All data are provided in the manuscript and supplementary materials.

## References

[1] Khair KU, Hasanuzzaman M (2021). Heavy metals-induced morphophysiological and biochemical changes in *Mentha piperita* L.. Approaches to the Remediation of Inorganic Pollutants.

[2] Fatima A, Hasanuzzaman M (2021). Heavy Metals Induced Physiological and Biochemical Changes in Fenugreek (*Trigonella foenum-graceum* L.). Approaches to the Remediation of Inorganic Pollutants.

[3] Sharma S, Nagpal AK, Kaur I (2018). Heavy metal contamination in soil, food crops and associated health risks for residents of Ropar wetland, Punjab, India and its environs. Food Chem..

[4] Abdel-Warith AA, Younis EMI, Al-Asgah NA, Rady AM, Allam HY (2020). Bioaccumulation of lead nitrate in tissues and its effects on hematological and biochemical parameters of *Clarias gariepinus*. Saudi J. Biol. Sci..

[5] Zubair M (2021). Heavy metals occurrence, seasonal variationand enrichment in Urban soils augmented with industrial waste. Pol. J. Environ. Stud..

[6] Ahmad W, Alharthy RD, Zubair M (2021). Toxic and heavy metals contamination assessment in soil and water to evaluate human health risk. Sci. Rep..

[7] Cai LM (2019). Heavy metal contamination and health risk assessment for children near a large Cu-smelter in central China. Sci. Total Environ..

[8] Kong X (2018). Heavy metal bioaccumulation in rice from a high geological background area in Guizhou Province, China. Int. J. Environ. Res. Public Health.

[9] Liu H (2021). Quantitative source apportionment, risk assessment and distribution of heavy metals in agricultural soils from southern Shandong Peninsula of China. Sci. Total Environ..

[10] Yang Q (2021). Distribution and secondary enrichment of heavy metal elements in karstic soils with high geochemical background in Guangxi, China. Chem. Geol..

[11] MEPPRC. Ministry of Environmental Protection of China And MLRPRC. http://www.mee.gov.cn/gkml/sthjbgw/qt/201404/W020140417558995804588.pdf (Ministry of Land and Resources of China, 2014) (Ministry of Ecology and Environment of China). Bulletin on National Survey of Soil Contamination in China.

[12] Wan M, Hu W, Wang H, Tian K, Huang B (2021). Comprehensive assessment of heavy metal risk in soil-crop systems along the Yangtze River in Nanjing, Southeast China. Sci. Total Environ..

[13] Xiang M (2021). Heavy metal contamination risk assessment and correlation analysis of heavy metal contents in soil and crops. Environ. Pollut..

[14] Guo B (2020). Health risk assessment of heavy metal pollution in a soil-rice system: a case study in the Jin-Qu Basin of China. Sci. Rep..

[15] Jiang Y (2017). Source apportionment and health risk assessment of heavy metals in soil for a township in Jiangsu Province, China. Chemosphere.

[16] Liu DX, Ma JH, Sun YL, Li YM (2016). Spatial distribution of soil magnetic susceptibility and correlation with heavy metal pollution in Kaifeng City, China. CATENA.

[17] Wang JR (2021). Source analysis of heavy metal pollution in agricultural soil irrigated with sewage in Wuqing, Tianjin. Sci. Rep..

[18] Wang S (2019). Spatial distribution and source apportionment of heavy metals in soil from a typical county-level city of Guangdong Province, China. Sci. Total Environ..

[19] Chai L (2021). Quantitative source apportionment of heavy metals in cultivated soil and associated model uncertainty. Ecotoxicol. Environ. Saf..

[20] Jiang HH (2020). An integrated approach to quantifying ecological and human health risks from different sources of soil heavy metals. Sci. Total Environ..

[21] Xiao R (2019). Accumulation, ecological-health risks assessment, and source apportionment of heavy metals in paddy soils: A case study in Hanzhong, Shaanxi, China. Environ. Pollut..

[22] Huang J (2018). A new exploration of health risk assessment quantification from sources of soil heavy metals under different land use. Environ. Pollut..

[23] Xie X, Liu Y, Qiu H, Yang X (2020). Quantifying ecological and human health risks of heavy metals from different sources in farmland soils within a typical mining and smelting industrial area. Environ. Geochem. Health.

[24] Yang SY (2019). An integrated analysis on source-exposure risk of heavy metals in agricultural soils near intense electronic waste recycling activities. Environ. Int..

[25] Wan F (2021). Pollution assessment, source identification, and health risks of heavy metals: A case study in a typical wheat–maize rotation area of eastern China. Environ. Geochem. Health.

[26] Dai JR (2011). Geochemical baselines and background values and element enrichment characteristics in soils in eastern Shandong Province. Geochimica.

[27] Muller G (1979). Index of geoaccumulation in sediments of the Rhine River. GeoJournal.

[28] Pang XG (2018). Background values of soil geochemistry in Shandong Province. Shandong Land Resour..

[29] Loska K, Wiechuła D, Korus I (2004). Metal contamination of farming soils affected by industry. Environ. Int..

[30] Zhao GL (2022). Source analysis and ecological risk assessment of heavy metals in farmland soils around heavy metal industry in Anxin County. Sci. Rep..

[31] Paatero P, Tapper U (1993). Analysis of different modes of factor analysis as least squares fit problems. Chemom. Intell. Lab. Syst..

[32] Hakanson L (1980). An ecological risk index for aquatic pollution control. A sedimentological approach. Water Res..

[33] CNEMC. *Natural Background Values of Soil Elements in China. Com/S0048-9697 (18)*. http://refhub.elsevier (Environmental Science, Beijing: China, 1990) (in Chinese) 34012-9/rf0040.

[34] Gan YD (2018). Source contribution analysis and collaborative assessment of heavy metals in vegetable-growing soils. J. Agric. Food Chem..

[35] Holmgren GGS, Meyer MW, Chaney RL, Daniels RB (1993). Cadmium, lead, zinc, copper, and nickel in agricultural soils of the United States of America. J. Environ. Qual..

[36] Song C, Yan Y, Rosado A, Zhang Z, Castellarin SD (2019). ABA alleviates uptake and accumulation of zinc in grapevine (*Vitis vinifera* L.) by inducing expression of ZIP and detoxification-related genes. Front. Plant Sci..

[37] Peng M (2020). Heavy metal and Pb isotopic compositions of soil and maize from a major agricultural area in Northeast China: Contamination assessment and source apportionment. J. Geochem. Explor..

[38] Zhang Q (2019). Spatial heterogeneity of heavy metal contamination in soils and plants in Hefei, China. Sci. Rep..

[39] Wang G (2017). Traffic-related trace elements in soils along six highway segments on the Tibetan Plateau: Influence factors and spatial variation. Sci. Total Environ..

[40] Zhang H, Wang Z, Zhang Y, Ding M, Li L (2015). Identification of traffic-related metals and the effects of different environments on their enrichment in roadside soils along the Qinghai-Tibet highway. Sci. Total Environ..

[41] Huang J (2019). Source apportionment and spatial and quantitative ecological risk assessment of heavy metals in soils from a typical Chinese agricultural county. Process Saf. Environ. Prot..

[42] Qin G (2021). Soil heavy metal pollution and food safety in China: Effects, sources and removing technology. Chemosphere.

[43] Ren WX (2014). Inventorying heavy metal pollution in redeveloped brownfield and its policy contribution: Case study from Tiexi District, Shenyang, China. Land Use Policy.

[44] Wang MT, Wang YW, Hu Y, Li Y (2016). Contribution analysis of the heavy metals in the soil from different sources to the biological toxicity based on the BP neural network model. J. Saf. Environ..

[45] Tang M, Lu G, Fan B, Xiang W, Bao Z (2021). Bioaccumulation and risk assessment of heavy metals in soil-crop systems in Liujiang karst area, Southwestern China. Environ. Sci. Pollut. Res. Int..

[46] Chen H (2016). Characteristics of heavy metal transfer and their influencing factors in different soil–crop systems of the industrialization region, China. Ecotoxicol. Environ. Saf..

[47] Yang S (2019). Status assessment and probabilistic health risk modeling of metals accumulation in agriculture soils across China: A synthesis. Environ. Int..

[48] Yang Q (2018). A review of soil heavy metal pollution from industrial and agricultural regions in China: Pollution and risk assessment. Sci. Total Environ..

[49] Kwong LH (2016). Hand- and object-mouthing of rural Bangladeshi children 3–18 months old. Int. J. Environ. Res. Public Health.

[50] EPAC. *Soil Environmental Quality Risk Control Standard for Soil Contamination of Agricultural Land (GB15618-2018)* (Environmental Protection Administration of China, 2018).

[51] Ministry of Land and Resources of the People's Republic of China. *Specification of land quality geochemical assessment (DZ/T 0295–2016).* (in Chinese).

[52] National Health and Family Planning Commission of the People’s Republic of China (NHFPCPRC) & China Food and Drug Administration (CFDA). *National Standard for Food Safety: Limit of Contaminants in Food; GB2762–2017; NHFPCPRC and CFDA: Beijing, China*, (2017). (in Chinese).

