# Supplementary material for: Heavy metal ecological-health risk assessment under wheat–maize rotation system in a high geological background area in eastern China
Source: Sci Rep. 2022 Oct 26;12:17912. doi: 10.1038/s41598-022-22608-z (PMC9606110; doi:10.1038/s41598-022-22608-z)
Supplement: Supplementary file 1 — Supplementary Information. [file 41598_2022_22608_MOESM1_ESM.docx]

**Table S1 Class standards for *I_geo_* of heavy metals**

| *I_geo_* | Class | Contamination assessment |
| --- | --- | --- |
| *I_geo_* ≤ 0 | Class 0 | Uncontaminated |
| 0 < *I_geo_* ≤ 1 | Class 1 | From uncontaminated to moderately contaminated |
| 1 < *I_geo_* ≤ 2 | Class 2 | Moderately contaminated |
| 2 < *I_geo_* ≤ 3 | Class 3 | From moderately to strongly contaminated |
| 3 < *I_geo_* ≤ 4 | Class 4 | Strongly contaminated |
| 4 < *I_geo_* ≤ 5 | Class 5 | From strongly to extremely contaminated |
| 5 < *I_geo_* | Class 6 | Extremely contaminated |

**Table S2 Grade standards for potential ecological risks of heavy metals**

| *E^i^_r_* | Grade |  | *RI* | Grade |
| --- | --- | --- | --- | --- |
| *E^i^_r_* ≤ 40 | Low |  | *RI* ≤ 150 | Low |
| 40 < *E^i^_r_* ≤ 80 | Moderate |  | 150 < *RI* ≤ 300 | Moderate |
| 80 < *E^i^_r_* ≤ 160 | Considerable |  | 300 < *RI* ≤ 600 | Considerable |
| 160 < *E^i^_r_* ≤ 320 | High |  | *RI* > 600 | Very high |
| *E^i^_r_* > 320 | Very high |  |  |  |

**Table S3 Input values of parameters for exposure dose assessment**

| Parameter | Description | Unit | Value | | References |
| --- | --- | --- | --- | --- | --- |
|  |  |  | Children | Adults |  |
| IR_ing_ | Ingestion rate of soil | mg day^-1^ | 200 | 100 | (USEPA 2001) |
| IR_inh_ | Inhalation rate of soil | m^3^ day^-1^ | 7.63 | 12.8 | (Xiao et al., 2015) |
| IR_diet_ | Ingestion rate of wheat | g day^-1^ | 73.64 | 140.2 | MEPPRC Exposure Factors Handbook of Chinese Population (2014) |
|  | Ingestion rate of maize | g day^-1^ | 16.68 | 23.6 |  |
| SA | Skin area available for soil contact | cm^2^ | 1600 | 4350 | Environmental site assessment guideline (2009) |
| AF | Soil-to-skin adherence factor | kg cm^-2^ day^-1^ | 0.2 | 0.7 | US EPA, 1993 |
| ABS | Absorption factor | unitless | 0.001 | 0.001 | USEPA, 2011 |
| PEF | Particle emission factor | m^3^ kg^-1^ | 1.36×10^9^ | 1.36×10^9^ | USEPA, 2001 |
| EF | Exposure frequency | day year^-1^ | 350 | 350 | USEPA, 2011 |
| ED | Exposure duration | year | 6 | 24 | USEPA, 2011 |
| BW | Body weight | kg | 15 | 70 | USEPA, 1999 |
| AT | Average time | day | 365ED | 365ED | USEPA, 1989 |

**Table S4 Values of reference dose (mg kg^-1^ day^-1^) for six heavy metals**

| Parameter | Cd | Cr | Pb | Cu | Zn | Ni | References |
| --- | --- | --- | --- | --- | --- | --- | --- |
| RfD for ingestion | 1.00E-03 | 1.50E+00 | 3.50E-03 | 4.00E-02 | 3.00E-01 | 2.00E-02 | USEPA, 2015; Yang et al., 2019 |
| RfD for dermal contact | 1.00E-05 | 6.00E-05 | 5.25E-04 | 1.20E-02 | 6.00E-02 | 5.40E-03 |  |
| RfD for inhalation | 1.00E-05 | 2.86E-05 | 3.50E-03 | 4.02E-02 | 3.00E-01 | 2.01E-02 | USEPA, 2011; Jiang et al., 2016 |
| RfD for diet | 1.00E-03 | 1.50E+00 | 3.50E-03 | 4.00E-02 | 3.00E-01 | 2.01E-02 | USEPA, 2010; Sharma et al., 2018 |

RfD, Reference dose

**Table S5 Statistical characteristics of heavy metals in crops**

| HM | Wheat (n = 68) | | | | |  |  | Maize (n = 68) | | | | |  | SV^a^ |
| --- | --- | --- | --- | --- | --- | --- | --- | --- | --- | --- | --- | --- | --- | --- |
|  | Mean  (mg/kg) | Min.  (mg/kg) | Max.  (mg/kg) | SD | CV% | ER% |  | Mean  (mg/kg) | Min.  (mg/kg) | Max.  (mg/kg) | SD | CV% | ER% |  |
| Cd | 0.038 | 0.013 | 0.095 | 0.02 | 48.07% | 0% |  | 0.009 | 0.003 | 0.067 | 0.01 | 109.05% | 0% | 0.1 |
| Cr | 0.13 | 0.04 | 0.49 | 0.08 | 64.01% | 0% |  | 0.56 | 0.13 | 1.59 | 0.33 | 58.53% | 11.76% | 1 |
| Cu | 6.26 | 3.81 | 9.72 | 1.47 | 23.46% | 0% |  | 2.03 | 1.21 | 4.69 | 0.58 | 28.60% | 0% | / |
| Ni | 0.54 | 0.08 | 1.51 | 0.32 | 60.09% | 0% |  | 0.72 | 0.22 | 2.18 | 0.35 | 48.62% | 0% | / |
| Pb | 0.06 | 0.04 | 0.09 | 0.01 | 18.73% | 0% |  | 0.07 | 0.01 | 0.31 | 0.05 | 72.58% | 4.41% | 0.2 |
| Zn | 42.96 | 18.97 | 92.95 | 19.27 | 44.85% | 0% |  | 18.97 | 12.32 | 27.73 | 3.02 | 15.91% | 0% | / |

HM = heavy metals; SD = standard deviation; CV = coefficient of variance; BV = background value; ER = excess rate; SV = standard value.

^a^National Health and Family Planning Commission of the People’s Republic of China (NHFPCPRC) and China Food and Drug Administration (CFDA). National Standard for Food Safety: Limit of Contaminants in Food; GB2762-2017.

**
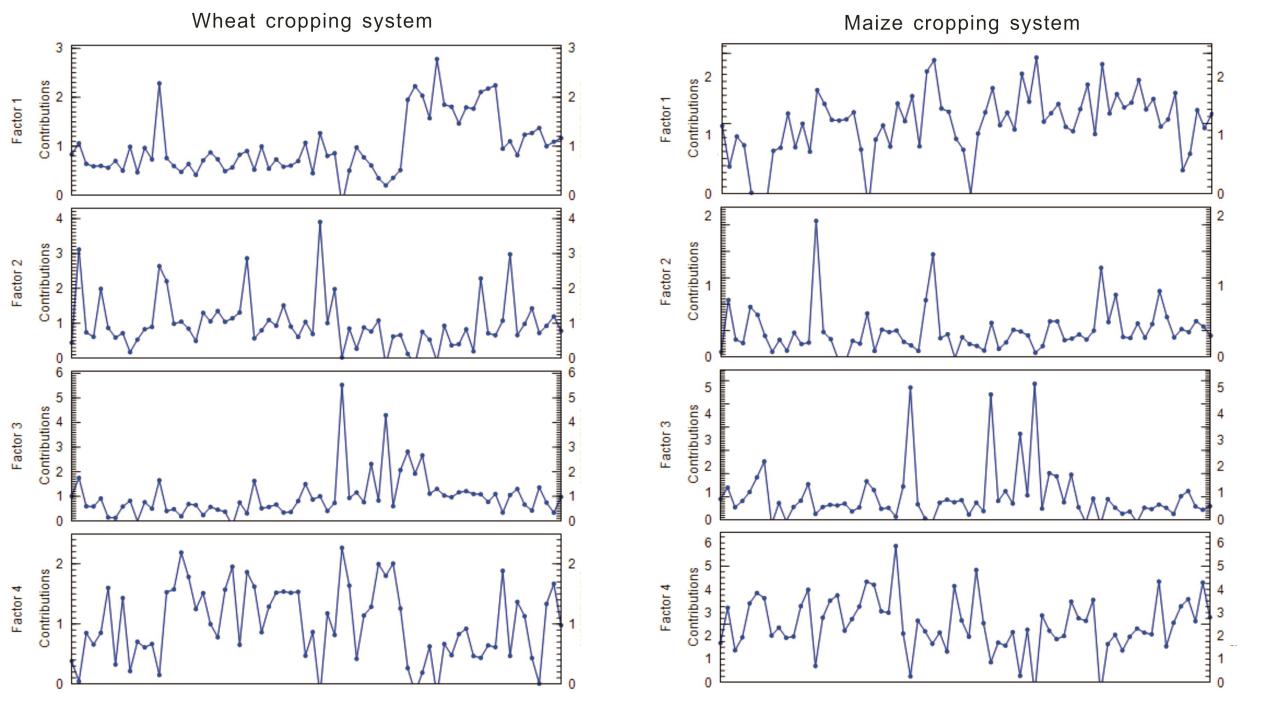
**

**Fig. S1.** **Normalized source contributions of heavy metals obtained by the PMF model (the average of normalized contributions to all sample sites=1).**

**
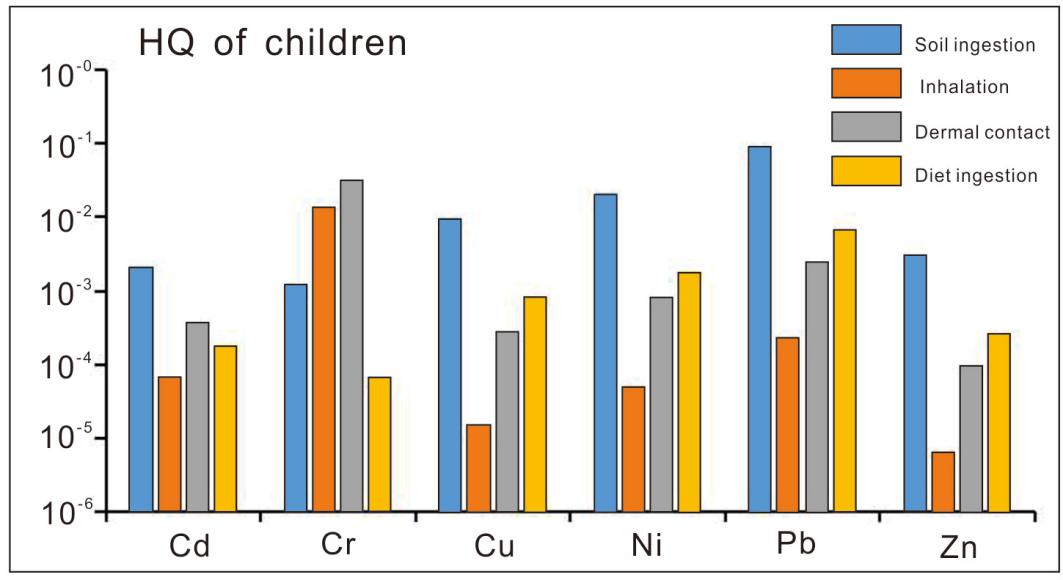
**

**
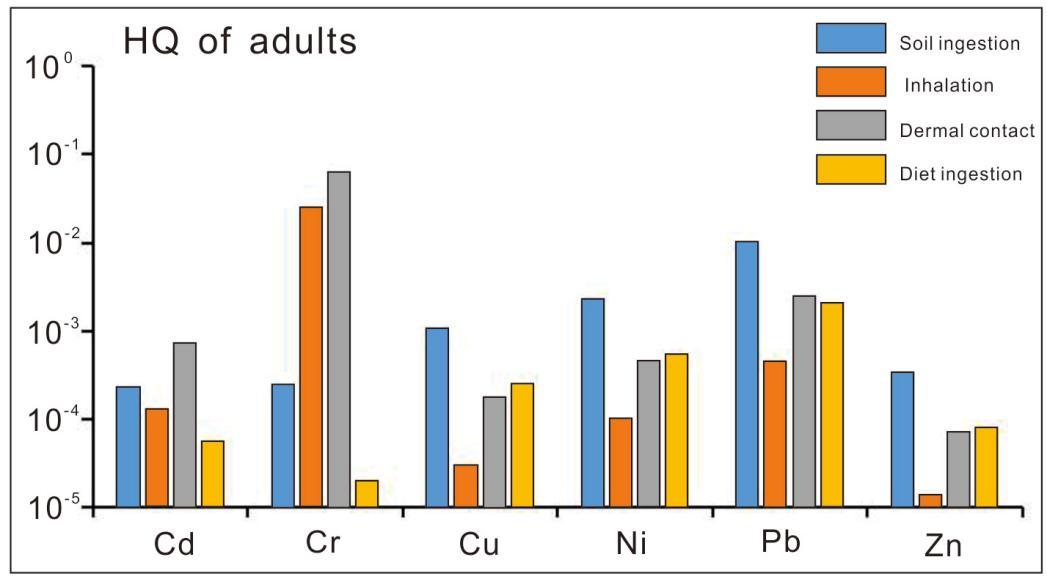
**

**Fig. S2. The hazard quotient (HQ) for heavy metals by four exposure pathways**


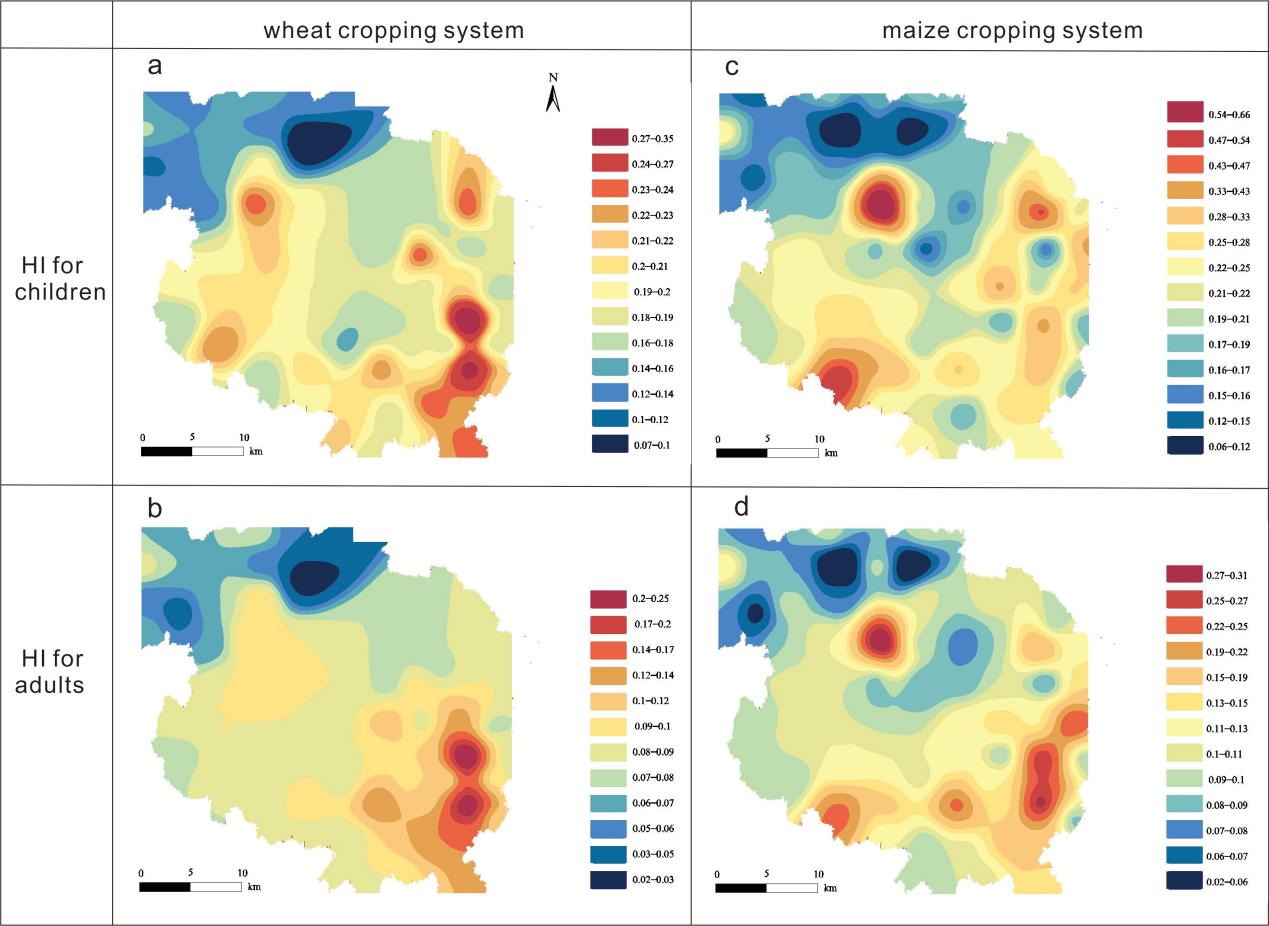


**Fig. S3. The spatial distribution of the hazard index (HI) for children and adults under wheat and maize cropping systems**

**References for Tables S3, S4 and S5**

National Health and Family Planning Commission of the People’s Republic of China (NHFPCPRC) and China Food and Drug Administration (CFDA). National Standard for Food Safety: Limit of Contaminants in Food; GB2762-2017; NHFPCPRC and CFDA: Beijing, China, 2017. (In Chinese)

Environmental site assessment guideline, 2009. DB11/T656-2009. (In Chinese).

Jiang, Y., Chao, S., Liu, J., Yang, Y., Chen, Y., Zhang, A., Cao, H., 2016. Source apportionment and health risk assessment of heavy metals in soil for a township in Jiangsu Province, China. Chemosphere 168, 1658-1668.

MEPPRC (Ministry of Environmental Protection of the People’s Republic of China) and MLRPRC (Ministry of Land and Resources of the People’s Republic of China), 2014. Bulletin on National Survey of Soil Contamination (in Chinese). Available at: <http://www.zhb.gov.cn/gkml/hbb/qt/201404/t20140417_270670.htm.>

Sharma S , Nagpal A K , Kaur I . Heavy metal contamination in soil, food crops and associated health risks for residents of Ropar wetland, Punjab, India and its environs[J]. Food Chemistry, 2018, 255(JUL.30):15-22.

USEPA (The United States Environmental Protection Agency), 1989. Risk assessment guidance for superfund. In: Human Health Evaluation Manual (part A). EPA/540/1-89/002. vol. 1. Environmental Protection Agency, Washington, DC.

USEPA (The United States Environmental Protection Agency), 1991. CASRN 7440-50-8US EPA, S., 1999. Soil Screening Guidance: Technical Background Document | Superfund | US EPA.

USEPA (United States Environmental Protection Agency ), 1993. Reference Dose (RfD): Description and Use in Health Risk Assessments. Background Document 1A. Integrated risk information system (IRIS).

USEPA,1997.ExposureFactorsHandbookEPA/600/

USEPA (The United States Environmental Protection Agency), 2001. Supplemental guidance for developing soil screening levels for superfund sites. OSWER9355.4-24. Office of Solid Waste and Emergency Response. US Environmental Protection Agency. Washington, DC.

USEPA (The United States Environmental Protection Agency), 2010. Risk-Based Concentration Table.

USEPA (The United States Environmental Protection Agency), 2011. Exposure Factors Handbook 2011 Edition (Final). U.S. Environmental Protection Agency, Washington, DC, EPA/600/R-09/052F.

USEPA (United States Environmental Protection Agency). (2015). Risk based screening table-generic, summary table. United States Environmental Protection Agency. URL http://www.epa.gov/risk/risk-based-screening-table-generic-tables Accessed 31.01. 2016.

Xiao Q , Zong Y , Lu S . Assessment of heavy metal pollution and human health risk in urban soils of steel industrial city (Anshan), Liaoning, Northeast China - ScienceDirect[J]. Ecotoxicology and Environmental Safety, 2015, 120:377-385.

Yang, S. Y., He, M. J., Zhi, Y. Y., Chang, S. X., Gu, B. J., Liu, X. M. (2019) An integrated analysis on source-exposure risk of heavy metals in agricultural soils near intense electronic waste recycling activities. Environment international, 133:105239. <https://doi.org/10.1016/j.envint.2019.105239>.
